# GA-Responsive Dwarfing Gene *Rht12* Affects the Developmental and Agronomic Traits in Common Bread Wheat

**DOI:** 10.1371/journal.pone.0062285

**Published:** 2013-04-26

**Authors:** Liang Chen, Andrew L. Phillips, Anthony G. Condon, Martin A. J. Parry, Yin-Gang Hu

**Affiliations:** 1 State Key Laboratory of Crop Stress Biology for Arid Areas and College of Agronomy, Northwest A&F University, Yangling, Shaanxi, China; 2 Department of Plant Biology and Crop Science, Rothamsted Research, Harpenden, Herts, United Kingdom; 3 CSIRO Plant Industry, Canberra, Australia; 4 Institute of Water Saving Agriculture in Arid Regions of China, Northwest A&F University, Yangling, Shaanxi, China; Instituto de Biología Molecular y Celular de Plantas, Spain

## Abstract

Opportunities exist for replacing reduced height (*Rht*) genes *Rht-B1b* and *Rht-D1b* with alternative dwarfing genes, such as the gibberellin-responsive gene *Rht12*, for bread wheat improvement. However, a comprehensive understanding of the effects and mode of action of *Rht12* is lacking. In the present study, the effects of *Rht12* were characterized by analyzing its effects on seeding vigour, seedling roots, leaf and stem morphology, spike development and carbohydrate assimilation and distribution. This was carried out in the four genotypes of F_2:3_ lines derived from a cross between Ningchun45 and Karcagi (12) in two experiments of autumn sowing and spring sowing. *Rht12* significantly decreased stem length (43%∼48% for peduncle) and leaf length (25%∼30% for flag leaf) while the thickness of the internode walls and width of the leaves were increased. Though the final plant stature was shortened (40%) by *Rht12*, the seedling vigour, especially coleoptile length and root traits at the seedling stage, were not affected adversely. *Rht12* elongated the duration of the spike development phase, improved the proportion of spike dry weight at anthesis and significantly increased floret fertility (14%) in the autumn sowing experiment. However, *Rht12* delayed anthesis date by around 5 days and even the dominant *Vrn-B1* allele could not compensate this negative effect. Additionally, grain size was reduced with the ability to support spike development after anthesis decreased in *Rht12* lines. Finally, grain yield was similar between the dwarf and tall lines in the autumn sowing experiment. Thus, *Rht12* could substantially reduce plant height without altering seeding vigour and significantly increase spikelet fertility in the favourable autumn sowing environment. The successful utilization of *Rht12* in breeding programs will require careful selection since it might delay ear emergence. Nonetheless, the potential exists for wheat improvement by using *Rht12*.

## Introduction

The introduction of semi-dwarfing genes into wheat was a major event in breeding high yielding varieties during the Green Revolution [1]. The greater grain yields were associated with improved lodging resistance and the resulting ability to tolerate higher rates of chemical fertilizers [2], and also with increased harvest index [3]. The gibberellin acid (GA) insensitive semi-dwarfing genes *Rht-B1b* (*Rht1*) and *Rht-D1b* (*Rht2*) are widely used to reduce plant height and increase grain yield in wheat breeding programs. *Rht-B1b* and *Rht-D1b* reduce stem internode length and therefore overall plant height by decreasing the sensitivity of vegetative and reproductive tissues to endogenous GA [4], [5]. However, reduced stature also contributes to reduced seedling vigour and coleoptile length and may reduce crop water-use efficiency [6]–[9], and performance in unfavourable environments [5], [10], [11]. Longer coleoptiles may permit crops to be sown at the optimal time to increase biomass and yield [12] while deep sowing of short coleoptile *Rht-B1b* and *Rht-D1b* alleles commonly results in fewer, later emerging seedlings with low relative growth rates, leaf area and biomass [13], and ultimately lower final biomass, fewer spikes and yield [13], [14]. But, in irrigated and fertilised environments, the height reduction associated with *Rht-B1b* and *Rht-D1b* may not be sufficient. Excessive height and severe lodging can occur in semi-dwarf varieties carrying these alleles [15], [16]. Further, genetic reductions in plant height of the *Rht-B1b*+*Rht-D1b* doubled-dwarfs usually produce much less biomass and result in slow development of seedling leaf area though they significantly reduced lodging and have a greater harvest index [7], [10], [17]. A range of dwarfing genes is needed at our disposal to achieve the appropriate height reduction for different environments [7]. The modest height-reducing gene *Rht8* may be suitable to reduce final plant height without compromising early plant growth. *Rht8* has been used in breeding programs in different environments [18], [19] as it has no effect on coleoptile length or seedling vigour [5], [20] and there is potential for developing shorter *Rht8*+*Rht-B1b*/*D1b* sesqui-dwarfs with reduced lodging susceptibility and without limiting crop establishment [21]. Preliminary evidence indicates the potential of several other GA-responsive dwarfing genes, including *Rht4*, *Rht5*, *Rht9*, *Rht12*, *Rht13* and *Rht14*, to reduce plant height without affecting seedling vigour [22]. It is currently not clear whether these alternative dwarfing genes can be used to improve wheat yield and lodging tolerance [23].

The life cycle of wheat can be divided into phases based on the main organs being differentiated [24]. The pre-anthesis late reproductive phase (from terminal spikelet initiation to anthesis) of stem elongation is particularly important for yield [25], [26] because the number of fertile florets at anthesis will be determined during this phase. Wheat yield can be improved through increasing the duration of the late reproductive phase by manipulation of spike developmental rates [27], [28]. The developmental phases prior to anthesis may be sensitive to the environment (photoperiod or temperature) that they encounter or the concentrations of different endogenous hormones (gibberellin, auxin or cytokinin) associated with development [29]–[32]. Indeed, several dwarfing genes are associated with lesions in GA biosynthesis or signalling [33], [34]. The possibility exists that the duration of spike development phases can be optimised using dwarfing genes, as done with genes affecting photoperiod or vernalization requirement [26], [35], [36]. Previous reports have shown that GA-insensitive dwarfing genes have no effect on the initiation of leaf and spikelet primordia at the shoot apex nor on the number of leaves and internodes. The primary effect of these dwarf genes on growth is to reduce the rate of leaf expansion, stem elongation and vegetative dry matter accumulation [37], [38]. However, the effects of GA-responsive dwarfing genes on plant growth and spike development have not been investigated and the potential of optimising the duration of late reproductive phase to improve ear fertility using GA-responsive dwarfing genes is unknown. Additionally, winter wheat requires several weeks at low temperature to flower. This process, vernalization, is controlled by three major genes, *Vrn-A1*, *Vrn-B1* and *Vrn-D1* which locate to the long arm of chromosomes 5A, 5B, and 5D, respectively [39], [40]. The spring habit alleles at these loci are dominant while recessive alleles at all three loci determine winter growth habit [41], [42]. The dominant *Vrn-A1* allele provides complete insensitivity to vernalization (achieving the double ridge stage without low temperature) whereas the dominant *Vrn-B1* and *Vrn-D1* alleles each provide a reduced vernalization requirement compared to winter alleles [43]. Thus, these genes can be used to modify the flowering time in wheat.

*Rht12*, a dominant GA-responsive dwarfing gene from the gamma ray induced mutant Karcagi 522M7K of winter wheat [44], has been shown to be located distally in the long arm of chromosome 5A, approximately 5.4 cM from locus Xwms291 [45], [46]. *Rht12* has been described as having few negative effects on yield components and, with a reduction of height by around 46% [44], it has a stronger effect on height than either *Rht-B1b* or *Rht-D1b.* Worland et al. [2] reported that *Rht12* reduced height without altering ear size and significantly increased spikelet fertility, but this was always accompanied by delayed ear emergence. There was no significant difference in early root growth and root architecture between *Rht12* dwarf lines and the control lines, but the effects on total root length were still unclear due to the different background or experimental methodology [47]. Recently, it was found that *Rht12* increased grain yield, harvest index and lodging resistance while reducing grain weight [21]. However, it is still difficult to evaluate the effects of *Rht12* comprehensively, which would include effects on vegetative organs, spike development or other agronomic traits. Moreover, *Rht12* has not been used in wheat breeding, and its full potential remains uncertain.

The objectives of this work were to analyse the effect of *Rht12* on leaf and stem morphology, phenological development up to anthesis and on agronomic traits and yield components. Due to the late ear emergence associated with the *Rht12* allele, the dominant *Vrn-B1* gene was introduced from a tall, spring-habit parent to analyse the effect of *Rht12* on spike development.

## Materials and Methods

### General Description

The experiments were carried out during the two growing seasons of 2010–2011 and 2011–2012 in the experimental field of the Institute of Water Saving Agriculture in Arid Regions of China, Northwest A&F University, Yangling, Shaanxi, China (34°17′ N, 108°04′ E, at an elevation of 506 m). To avoid water stress, supplemental irrigation was provided as needed. Weeds were manually removed where necessary, and fungicides and insecticides were applied to prevent diseases and insect damage. Weather data were recorded at an automated weather station at the site.

### Plant Material

A cross was made using Ningchun 45 as the female and Karcagi (12) as pollen donor in May, 2009. Wheat seeds of Karcagi (12) (*Triticum aestivum* L.), a gamma ray-induced mutant carrying the dominant GA-responsive dwarfing gene *Rht12* and strong winter habit as detected with the recessive loci for all three *Vrn-1* genes, were generously provided by the Australian Winter Cereal Collection. Ningchun 45 (*Triticum aestivum* L.), is a tall Chinese spring wheat cultivar widely used in the semi-arid areas of Northwest Spring Wheat Region of China, which carries the dominant vernalization gene *Vrn-B1* and lacks any known dwarfing genes as detected by molecular markers.

The F_2_ population was sown as spaced plants in the field in October, 2010. The individuals of the F_2_ population were numbered, the plant height and other agronomic traits of each individual were recorded, and the presence or absence of the loci for the dwarfing gene *Rht12* and vernalization gene *Vrn-B1* in each numbered individual was determined using the corresponding molecular markers (see below for details). The individuals with the four kinds of homozygous genotypes of *Rht12Rht12Vrn-B1Vrn-B1* (abbreviated as RRBB), *Rht12Rht12vrn-B1vrn-B1* (RRbb), *rht12rht12Vrn-B1Vrn-B1* (rrBB) and *rht12rht12vrn-B1vrn-B1* (rrbb) were then selected and used to develop the F_2:3_ lines for further analysis.

The two parents and 57 F_2:3_ homozygous lines were used in experiments to evaluate the effects of dwarfing gene *Rht12* in the 2011–2012 growing season. There were two sowing dates: October 6, 2011 (Autumn Sowing, AS) and February 6, 2012 (Spring Sowing, SS). Among those F_2:3_ homozygous lines, 15, 13, 17 and 12 lines had the genotypes *Rht12Rht12Vrn-B1Vrn-B1*, *Rht12Rht12vrn-B1vrn-B1*, *rht12rht12Vrn-B1Vrn-B1*, and *rht12rht12vrn-B1vrn-B1*, respectively, as identified in the F_2_ population. The lines and parents were sown in plots of four rows 2 m long and 25 cm apart, with seeds spaced 5 cm apart within rows. The parents and the 28 dwarf and 29 tall F_2:3_ lines were randomly arranged to avoid competitive effects with two replications.

### Genotyping of *Rht12* and *Vrn-B1*

The molecular markers WMS291 (Xgwm291) and Intr1-B-F/Intr1-B-R3/Intr1-B-R4 were used to determine the genotypes of individuals for the presence or absence of the dwarfing gene *Rht12* [46] and the vernalization gene *Vrn-B1* [48] in the individual plants of the F_2_ population, respectively.

Genomic DNA from young leaves was isolated from the two parents and each F_2_ individual using the CTAB method [49]. DNA concentrations were measured using a spectrophotometer and normalized to 50 ng/µL. The PCR protocols for the three markers were as described [46], [48]. The PCR profiles were: an initial denaturation at 95°C for 2 min was followed by 40 cycles at 94°C for 20 s, 55°C, 63°C and 58°C (for the three markers, respectively) for 30 s, 72°C for 90 s and a final extension of 72°C for 5 min. PCR products for SSR marker Xgwm291 were visualized on a 8% polyacrylamide gel (19∶1 of Acrylamide to bis ratio) in 1×TBE buffer at 250V for 1.5 h, then stained by silver buffer using a modified procedure described previously [50]. PCR products for Intr1-B-F and Intr1-B-R3 or R4 were separated on 1% agarose gels with 1×TAE buffer at 115 V for 40 min, stained in an ethidium bromide buffer for 15 min, and then visualized by Gel Doc XR (BioRad Laboratories, Inc.).

### Coleoptile Length and Seedling Root Traits

Coleoptile length from the seed to the tip of the coleoptile was measured with a ruler after germination in a darkened growth chamber at 20°C after 200°Cd using the method described [5], [51]. Seedling root growth characteristics were assessed using a ‘Cigar’ method as follows. Five grains of each line were arranged in a line (3 cm from the upper edge) on a 32×29 cm germination paper (Anchor, USA), and then rolled as a cigar with the seeds at the top. Then the cigar was stood vertically in a tank (50×35×30 cm) containing Hoagland solution (10 cm depth) at 20°C until 200°Cd. The number of seminal roots, maximum root length, and total root length were measured. Root and shoot dry mass were also investigated after drying at 60°C for 72 h. Seedling vigour was evaluated as the area of the first two leaves, coleoptile length and the seedling root length.

### Spike Development and Fertility

Spike differentiation was investigated on three randomly selected plants from each field plot. Beginning from the three leaf stage (Z12), plants were sampled every 3 days and the main shoot was dissected to determine the timing of the double ridge formation (DR) and the terminal spikelet initiation (TS) in the apical meristem, as described by [52] using a digital Stereo Microscope (Nikon, SMZ1500). The timing of other stages of spike differentiation was also recorded successively. Pictures were taken using a digital camera linked to the microscope. The timing of heading (Z55) and anthesis (Z65) was visually determined when 50% of the plants per plot had reached these stages.

At anthesis, five plants in the central row of each plot were harvested and the number of fertile florets in the main shoot spike was counted before drying. Florets were considered fertile when the stigmatic branches were spread wide, with either pollen grains present on them or with green anthers [53].

### Carbohydrate Assimilation and Distribution

After investigation of fertility, the five plants harvested at anthesis were partitioned to investigate the dry weight of the main shoot (PDM), dry weight of the main shoot spike (SDM), dry weight of tiller spikes (SDT) and the total dry matter excluding roots (TDP). The partitioning of total dry matter to reproductive organs (dry weight of total spike including main shoot and tiller spikes) and the spike to stem ratio in main shoots were then calculated. The number of green leaves on the main shoot was counted from the flag leaf down and included fractions of leaves that were partially yellow [54].

Also from these samples, the dry weights of flag leaf, the second leaf, the third leaf, the peduncle, the fifth internode, the fourth internode, the basal three internodes on the main shoot stems and leaves including leaf sheaths were determined. Samples were also collected at the 14th, 18th, 22nd and 30th day after anthesis as described above to analyse the dynamic changes of the dry weight of spike, stem and leaf of the main shoot between different genotypes. All samples were oven-dried at 60°C for 72 h and weighed separately [26], [55].

### Plant Height, Leaf and Internode Character

Plant height was determined at maturity as the distance from the soil surface to the top of the ear (awns excluded) of ten plants for each plot. Seedling height was measured as the distance from the soil surface to the ligule of the last fully emerged leaf of ten plants for each plot and was used to analyze the growth rate of different lines (capacity to produce biomass) weekly from Z12 until heading. At the three leaf stage (Z13) [54], five plants per plot were randomly selected and tagged, and the number of leaves emerging on the main shoot was noted two or three times a week until anthesis [56]. The length and width of each leaf were measured when the leaf was fully elongated.

All of the genotypes have six internodes, the first internode of more than 1 cm was defined as internode1, although internodes shorter than 1 cm were occasionally observed in some individuals [57]. Subsequent internodes up the stem were numbered as 2, 3, 4, 5, and 6 (peduncle), respectively. The lengths of internodes (mm) were measured from the mid-point of their subtending nodes. Stem diameter (mm) was measured at the middle of each internode using digital calipers (TESA Etalon). Internodes were then cut at their centre point and digital calipers were used to measure the stem wall thickness (mm). Two measurements of stem wall thickness were taken on opposite sides of the stem from which a mean value was calculated [57]. Characters of each internode were measured when they fully developed.

Lodging was scored at maturity from the fraction of the area affected and the severity of lodging in those areas noted, on a scale of 0 for a standing crop to 90 for a crop flat on the ground [54].

### Yield Components and Yield

The main shoot ears of ten plants of each line at maturity were measured for assessment of spike length, spikelets per spike, grains per spike, total grains in the uppermost three spikelets and in the bottom three spikelets of the spike. The fertile shoots per plant were also measured in the same plants.

Due to the frequent sampling prior to harvest and the limited number of plants in each plot (seeds from individual F_2_ plants were limited), only 25 to 40 plants remained in each plot at harvest. Plants were hand-cut at ground level and the number of plants harvested in each plot was recorded. The total above-ground dry biomass of each line was determined before threshing, and then the average biomass per plant, average yield per plant, harvest index (calculated as the ratio of grain to total above-ground biomass) and 1000-grain weight were determined.

### Data Analysis

For each parameter measured, the mean value for each line (15 RRBB, 13 RRbb, 17 rrBB and 12 rrbb lines) was calculated and statistical evaluation of the data was carried out by ANOVA analysis with multiple comparisons (LSD test at the 0.05 level) using the statistical package SPSS18.0. Association analysis was also made with SPSS18.0. Whenever thermal time was used to measure developmental progress, 0°C was chosen as base temperature.

## Results

### The Pleiotropic Effects of *Rht12* in F_2_ Progeny

Among the F_2_ segregating population, 228 plants were genotyped using the SSR markers linked with *Rht12* and *Vrn-B1*, respectively. Individuals homozygous at those two loci were selected and classified into four groups. Thus the F_2_ individuals composing 15 with RRBB (*Rht12Rht12Vrn-B1Vrn-B1*), 13 with RRbb (*Rht12Rht12vrn-B1vrn-B1*), 17 with rrBB (*rht12rht12Vrn-B1Vrn-B1*) and 12 with rrbb (*rht12rht12vrn-B1vrn-B1*) were identified.

All progenies segregated into two distinct height classes, due to the strong effects of *Rht12*, and the homozygous recessive *rht12* progeny could be clearly identified by their tall stature. The primary and pleiotropic effects of *Rht12* are shown in Table 1. Plant height was significantly decreased from an average of 106 cm in the rr genotypes to 76 cm in the RR genotypes; a reduction of 28% in plant height was thus observed. The individuals with heterozygous genotype (Rr) were approximately 4 cm taller than RR (data not shown). The spike length, number of spikelets per spike and 1000-grain weight were also reduced after introducing the dwarfing gene *Rht12*. Whereas, the grain number per spike, the effective spike number, and the fertility of the top three spikelets were increased in the homozygous dwarf plants (Table 1). Moreover, the ear emergence time and flowering time of the homozygous dwarf individuals were significantly delayed, for flowering by an average of 4 days (Table 1), but with some plants by as much as 10 days compared with the tall plants.

**Table 1:** Effects of *Rht12* on plant height, spike traits and yield components in the F_2_ progeny.

Genotype/variety	Plant height(cm)	Spike length(cm)	No. of spikeletsspike^−1^	No. of grainsin thetop three spikelets	No. of grainsin thebasal three spikelets	Grain numberspike^−1^	1000-grainweight (g)	Effective Tiller number	SW-AN* (°C d)
RRBB	75.5±6.97b	14.2±0.89b	20.5±1.01ab	5.5±0.23a	4.2±0.35a	42.4±4.65a	37.6±3.41b	12.4±2.94a	216.0 (1585)a
RRbb	76.4±7.43b	13.8±0.90b	19.9±1.12b	5.4±0.25a	3.9±0.40a	41.6±4.87a	36.3±2.31b	12.0±3.21a	216.3 (1592)a
rrBB	105.2±4.62a	15.1±0.82a	21.1±0.74a	4.4±0.21b	4.2±0.29a	39.6±4.29b	45.6±3.75a	8.7±2.68b	212.0(1479)b
Rrbb	107.2±4.61a	14.9±0.76ab	20.6±1.04ab	4.1±0.14b	4.3±0.24a	38.5±3.85b	44.5±3.29a	8.6±2.56b	212.5 (1500)b
Karcagi	73.0±2.48	13.7±0.66	20.0±0.77	6.2±0.15	3.9±0.23	43.6±3.29	33.9±1.39	12.7±1.43	221.0 (1633)
Nchun45	103.0±1.94	15.4±0.58	21.6±0.70	5.0±0.19	4.9±0.19	44.5±4.11	43.2±2.40	9.0±1.16	211.7 (1471)

* Data are the duration days (d) with calculated thermal time (°C d) in parenthesis; SW: sowing date; AN: anthesis date. Karcagi and Nchun45 represent the two parents Karcagi (12) and Ningchun45, respectively. Data are means ±SD (standard deviation) of each genotype. Data of the two parents were not considered in the statistical significance testing. Different letters within columns indicate statistically significant differences (*P*<0.05).

### Seedling Vigour and Seedling Root Characters

In the F_2:3_ lines, no significant difference was observed for the length or width of the first leaf between the tall and dwarf groups, while the length of the second leaf of the dwarf group was shorter than that of the tall group (with a 1.6 cm reduction) (Table 2). The total area of the first two leaves in the dwarf group were less than that of the tall group by 1.1 cm^2^ (9%) and 0.55 cm^2^ (11%) in the AS and SS experiments, respectively (Table 2). Although the area of individual leaves at seedling stage of the dwarf group was slightly reduced, the emergence of new leaves was advanced 3 days earlier than the tall group (data not shown), which may benefit seedling growth. However, genotype rrBB achieved the largest leaf area while RRbb had the smallest leaf area in both the AS and SS experiments (Table 2), indicating that the combination of RR and bb may affect seedling leaf growth. Additionally, no significant difference in coleoptile length was observed between the dwarf and tall groups (Table 3), suggesting that *Rht12* may confer a semi-dwarf height in wheat, while allowing no reduction in coleoptile length.

**Table 2:** Seedling vigour of different groups of the F_2:3_ lines in the autumn-sown (AS) and spring-sown (SS) experiments.

Experiment	Genotype/variety	Length of the 1^st^ leaf (cm)	Width of the 1^st^ leaf (cm)	Area of the 1^st^ leaf (cm^2^)	Length of the 2^nd^ leaf (cm)	Width of the 2^nd^ leaf (cm)	Area of the 2^nd^ leaf (cm^2^)	Total area of two leaves (cm^2^)
AS	RRBB	10.1±0.31a	0.5±0.04a	4.0±0.58a	13.0±0.70b	0.6±0.04a	6.2±0.81b	10.2±1.36b
	RRbb	10.0±0.22a	0.5±0.04a	4.0±0.47a	12.8±0.72b	0.6±0.04a	6.1±0.83b	10.1±1.23b
	rrBB	10.5±0.54a	0.5±0.04a	4.2±0.66a	15.0±1.44 a	0.6±0.04a	7.2±1.24a	11.4±1.61a
	rrbb	10.2±0.52a	0.5±0.03a	4.1±0.59a	14.4±1.32a	0.6±0.03a	6.9±1.18a	11.0±1.32ab
	Karcagi	9.0±0.19	0.5±0.04	3.6±0.43	11.0±0.35	0.6±0.04	5.3±0.54	8.9±0.89
	Nchun45	12.3±0.39	0.5±0.04	4.9±0.54	15.9±0.70	0.6±0.04	7.6±0.77	12.5±1.36
SS	RRBB	8.5±0.85a	0.3±0.04a	2.0±0.97a	10.4±0.58ab	0.3±0.04a	2.5±0.89a	4.5±0.93a
	RRbb	8.2±0.87a	0.3±0.04a	2.0±1.05a	9.9±0. 47b	0.3±0.04a	2.4±0.72a	4.4±0.90a
	rrBB	9.0±0.82a	0.3±0.04a	2.2±0.91a	11.6±1.15a	0.3±0.04a	2.8±1.28a	5.0±1.57a
	rrbb	8.7±0.80a	0.3±0.03a	2.1±090a	11.3±0.87a	0.3±0.03a	2.7±0.94a	4.8±1.14a
	Karcagi	7.1±0.31	0.3±0.04	1.7±0.43	8.3±0.35	0.3±0.04	2.0±0.58	3.7±0.89
	Nchun45	9.5±0.35	0.3±0.04	2.3±0.58	12.2±0.58	0.4±0.04	3.9±0.77	6.2±1.01

All data are means ±SD of each genotype. Data of the two parents were not considered in the statistical significance testing. Different letters within columns indicate statistically significant differences (*P*<0.05).

**Table 3:** Effects of *Rht12* on coleoptile length and root traits of different groups of the F_2:3_ lines at the seedling stage.

Genotype	Coleoptile length (cm)	Number of seminal roots	Maximum root length* (cm)	Total root length (cm)	Root dry mass (mg)	Shoot dry mass (mg)	Root to shoot Ratio of dry mass
RRBB	4.87±0.56a	3.0±0.08b	23.25±1.90a	57.46±3.29b	5.57±0.35b	11.32±1.20b	0.49±0.01a
RRbb	4.96±0.52a	3.0±0.14b	22.97±1.62a	57.14±2.74b	5.51±0.29b	11.15±1.26b	0.49±0.02a
rrBB	5.20±0.49a	5.1±0.16a	20.42±1.44b	63.39±4.33a	6.28±0.37a	14.29±1.81a	0.44±0.02b
rrbb	5.13±0.60a	5.0±0.21a	20.65±1.52b	63.20±3.15 a	6.26±0.24a	14.33±1.66a	0.44±0.01b
Karcagi(12)	4.1±0.22	3.0±0.08	22.47±0.93	49.67±2.52	4.64±0.23	10.43±0.97	0.44±0.01
Nchun45	6.2±0.29	5.3±0.08	24.28±1.98	68.42±4.80	7.23±0.50	14.58±2.05	0.50±0.03

* Mean value of the longest root length in each set of lines. Data are means ±SD of each genotype. Data of the two parents were not considered in the statistical significance testing. Different letters within columns indicate statistically significant differences (*P*<0.05).

**Figure 1:**
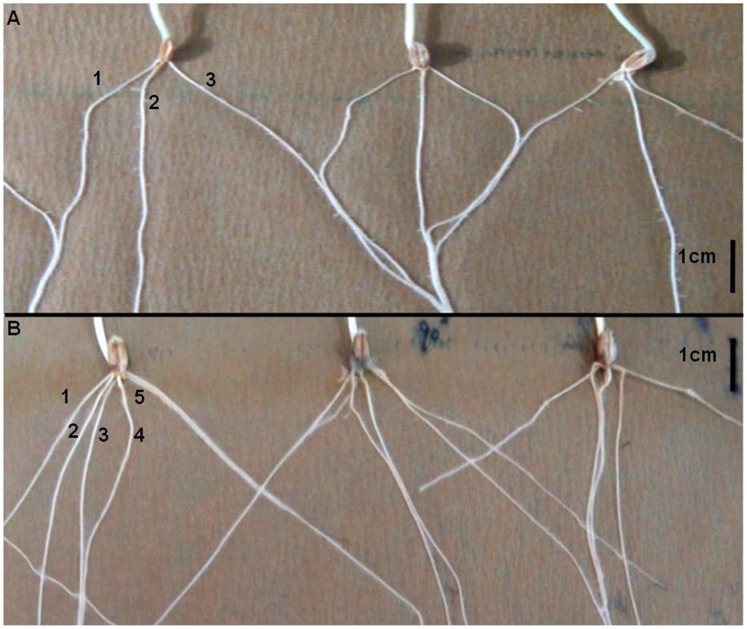
Seminal root morphology of the dwarf and tall lines at the seedling stage determined by the Cigar method. Panel A shows the dwarf lines and B shows the tall lines. The tall lines always have two more seminal roots than the dwarf lines (*P*<0.05).

Based on the ‘Cigar’ test for the root characters at the seedling stage, the total number of seminal roots was significantly decreased in the dwarf group (2 roots fewer) (Fig. 1), while the total root length in the dwarf group was reduced by 6 cm (9%) compared to that of the tall group (Table 3). The maximum root length of the dwarf group was 3 cm (15%) longer than that of the tall group, which may be beneficial for water acquisition in deeper soil in dry environments, assuming this seedling trait is expressed in adult plants.

The difference between the total root length of the tall and dwarf genotypes was mainly determined by the difference in total number of roots and not by the root elongation rate. The root dry mass was decreased by 0.7 mg (11%) in the dwarf lines. While, the dry mass ratio of root to shoot of the dwarf lines (0.49) was significantly greater than that of the tall lines (0.44). It suggested that *Rht12* did not greatly affect the seedling root growth although it significantly reduced the total number of roots. There was no significant difference in root architecture between BB and bb genotypes.

### Spike Development, Spike Dry Weight at Anthesis and Floret Fertility

**Figure 2:**
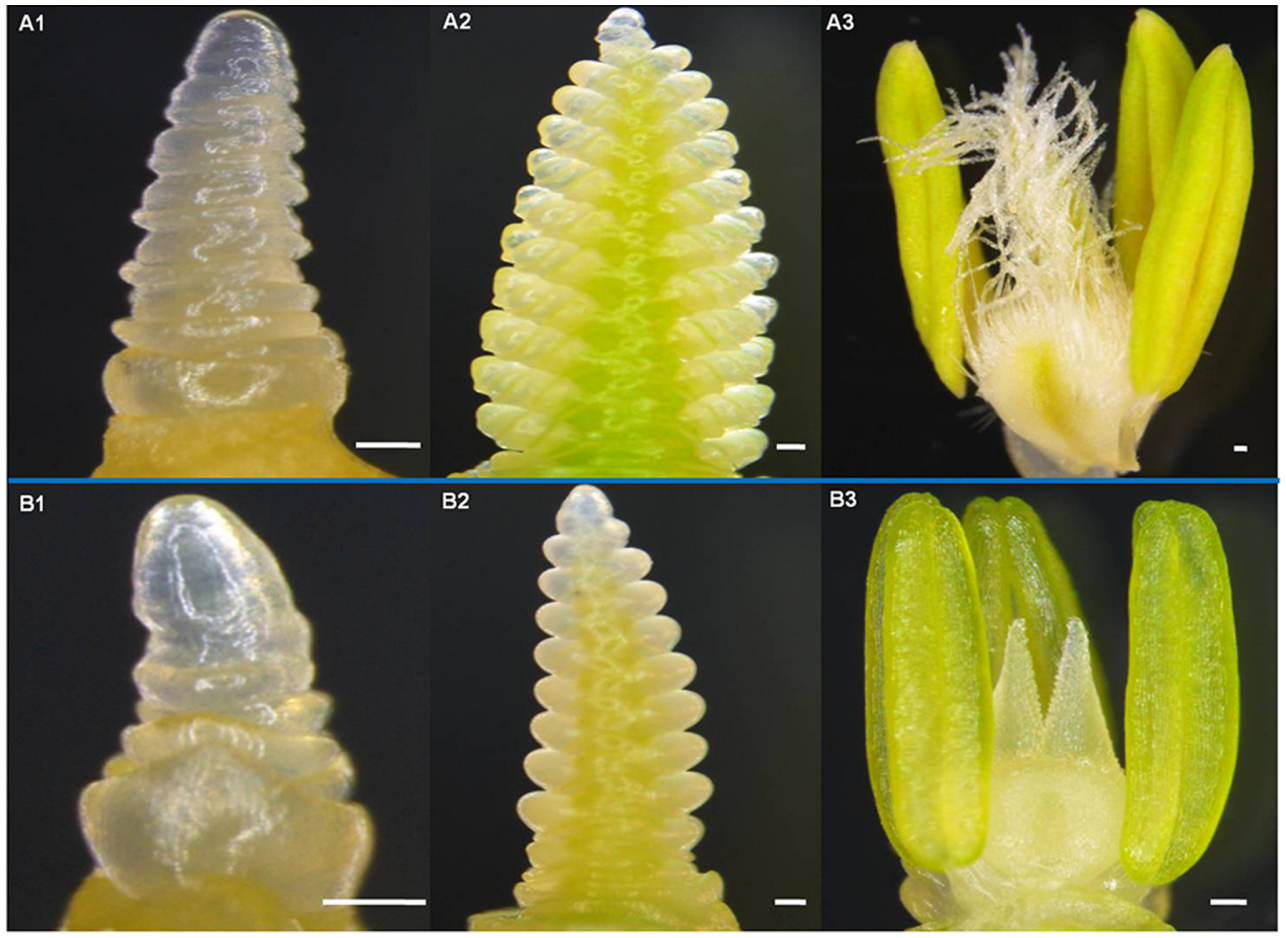
Spike development of the tall (rrBB, A1–A3) and dwarf (RRBB, B1–B3) genotypes in the AS experiment. A1 and B1 show spike development at the early double ridge stage of tall genotypes and at metaphase of single ridge stage of the dwarf genotypes at 630°Cd (75d after sowing), respectively; A2 and B2 show spike development at terminal spikelet initiation stage of the tall genotypes and at anaphase of double ridge stage of the dwarf genotypes at 770°Cd (165d after sowing), respectively; A3 and B3 show the floret morphology at yellow anther stage/anthesis of the tall genotypes and at green anther stage of the dwarf genotypes at 1430°Cd (209d after sowing), respectively. Scale bars = 100 µm.

Spike development was significantly delayed and the duration of the spike development phase was elongated in the *Rht12* dwarf lines compared to that of tall lines under both AS and SS experiments (Table 4). In the AS experiment, spike development of the dwarf lines with *Rht12* either with *Vrn-B1* or *vrn-B1* was delayed by 16 days to reach the double ridge stage, which meant that an additional thermal time of 45°Cd was needed compared to the tall lines with the winter growth habit. The effect of the dominant vernalization gene *Vrn-B1* was masked in the dwarf lines, while in the tall lines without *Rht12*, the lines with *Vrn-B1* reached double ridge stage about 60 days (30°Cd) earlier than those with *vrn-B1*. However, the anthesis date of the dwarf lines was about 5 days (110°Cd) later than that of the tall lines (Table 4 and Fig. 2). In the SS experiment, the RRBB lines required an additional 9 days (140°Cd) to reach the double ridge stage compared to the rrBB lines and needed a further 6 days (135°Cd) to reach anthesis compared to the tall lines. Whereas, rrBB lines reached double ridge stage about 5 days (60°Cd) faster than rrbb lines (Table 4).

**Table 4:** Duration days (d) and thermal time (°C d) of different pre-anthesis developmental phases of different groups of the F_2:3_ lines in the autumn-sown (AS) and spring-sown (SS) experiments.

Experiment	Genotype/variety	SW-AN	SW-DR	DR-TS	TS-AN	Total leaf number
AS	RRBB	214.0(1555.8)a	153.0(702.2)a	24.0(160.1)a	37.0(693.5)a	14.1a
	RRbb	215.5(1584.6)a	154.0(705.0)a	24.5(168.3)a	37.0(711.3)a	14.2a
	rrBB	209.0(1437.7)b	73.0(629.0)b	94.0(144.3)b	42.0(664.4)b	12.4c
	rrbb	210.5(1468.4)b	137.0(658.2)d	33.0(136.2)a	40.5(674.0)b	13.6b
	Karcagi	220.0(1661.6)	162.0(747.1)	22.0(194.0)	36.0(720.5)	16.0
	Nchun45	210.0(1458.9)	65.0(615.7)	102.0(156.1)	48.0(687.1)	11.0
SS	RRBB	114.0(1367.5)a	74.0(566.0)a	12.0(217.2)a	28.0(543.3)b	11.0a
	RRbb	115.0(1385.1)a	75.0(582.1)a	12.0(234.0)a	28.0(566.0)b	10.9a
	rrBB	108.0(1231.6)b	65.0(428.0)c	11.0(184.4)b	32.0(619.2)a	9.2c
	rrbb	109.0(1266.6)b	69.0(485.3)b	10.0(181.0)b	30.0(600.3)a	10.1b
	Karcagi	121.0(1471.5)	79.0(650.4)	13.0(249.0)	29.0(572.1)	12.0
	Nchun45	106.0(1192.8)	64.0(413.5)	11.0(191.2)	31.0(588.1)	9.0

Data are the duration days (d) with calculated thermal time (°C d) in parenthesis; SW: sowing date, DR: double ridge formation date, TS: terminal spikelet initiation date, AN: anthesis date. All data are means of each genotype. Statistical analysis was carried out using the thermal time. Data of the two parents were not considered in the statistical significance testing. Different letters within columns indicate statistically significant differences (*P*<0.05).

In both AS and SS experiments, there was no significant difference between RRBB and RRbb in spike development, suggesting that the dwarfing *Rht12* allele might have an epistatic effect on *Vrn-B1*. The tall spring lines (rrBB) developed faster than the tall winter lines (rrbb) before terminal spikelet stage while they had no difference on flowing time (Table 4). This indicated that genotypes having dominant *Vrn-B1* needed less time to undergo vernalization in tall lines. However, the effects of *Vrn-B1* on plant development were not strong enough to promote early flowering in either tall or dwarf genotypes.

In the AS experiment, spike lengths in RRBB and RRbb groups were significantly decreased (7%) compared with rrBB, while there was no significant difference observed with rrbb (Table 5). The number of spikelets per spike in RRbb was significantly reduced (3%) compared to that in rrBB and rrbb groups, while there was no significant difference between RRBB and rrBB/rrbb. The rrBB lines showed the largest number of spikelets per spike while RRbb had the smallest (Table 5). The dominant *Vrn-B1* allele likely plays a major role in determining spikelet number in this population and the combination of rr and BB produces more spikelets per spike. This phenomenon was also observed in the SS experiment. But, total florets per spike of RRBB, RRbb and rrbb were significantly decreased compared with that of rrBB in both experiments. This indicates that the BB allele apparently has an effect on initiation of florets in tall plants.

**Table 5:** Effects of *Rht12* on the spike characters and fertility of different groups of the F_2:3_ lines in the autumn-sown (AS) and spring-sown (SS) experiments.

Experiment	Genotype/variety	Spike length(cm)	Spikeletsspike^−1^	Florets initiatedspike^−1^	Fertile floretsspike^−1^	Fertile florets inthe top threespikelet	Fertility*
AS	RRBB	13.6±1.05b	20.2±0.89ab	197.0±16.42b	48.8±3.60a	6.1±0.19a	0.25±0.01a
	RRbb	13.5±0.71b	19.9±0.79b	194.2±14.78b	49.3±3.52a	6.3±0.32a	0.25±0.01a
	rrBB	14.6±0.99a	20.7±1.15a	202.9±17.03a	45.2±3.77b	4.2±0.33b	0.22±0.01b
	rrbb	14.1a±0.73a	20.4±1.07a	198.6±15.28b	44.1±4.02b	4.3±0.17b	0.22±0.01b
	Karcagi	12.4±0.54	18.7±0.66	169.9±13.71	46.7±3.82	7.3±0.19	0.27±0.01
	Nchun45	14.7±1.01	21.5±1.39	204.5±18.05	50.1±4.76	5.2±0.08	0.24±0.01
SS	RRBB	14.4±0.97ab	20.8±1.20b	201.8±19.83c	39.8±2.01b	4.8±0.12a	0.20±0.01b
	RRbb	14.0±1.19b	20.4±1.26b	198.5±16.87c	37.7±1.98b	4.4±0.18ab	0.19±0.01b
	rrBB	15.3±0.78a	21.6±1.11a	215.0±20.86a	51.0±2.60a	4.1±0.08b	0.24±0.02a
	rrbb	15.0±0.83a	21.4±1.04a	210.0±18.46b	49.9±1.97a	4.3±0.10ab	0.24±0.01a
	Karcagi	12.7±0.46	19.2±0.62	181.3±15.57	35.3±1.28	4.3±0.08	0.19±0.01
	Nchun45	15.3±0.77	21.9±1.32	220.2±16.46	55.4±3.37	4.1±0.19	0.25±0.02

* Fertility is estimated as the ratio of fertility florets to total floret per spike of the main shoot spike. All data are means ±SD of each genotype. Data of the two parents were not considered in the statistical significance testing. Different letters within columns indicate statistically significant differences (*P*<0.05).

In the AS experiment, *Rht12* significantly increased floret fertility (14%) and produced more fertile florets (10%) compared with the tall genotypes (Table 5). Correlation analysis showed that the elongated duration of the late reproduction phase pre-anthesis (thermal time accumulated) associated with *Rht12* was highly correlated with the increased number of fertile florets, and the duration of TS-AN explained almost 87% (R^2^ = 0.865) of variation of the ear fertility. However, possibly due to slow development of the vegetative phase (SW-DR) and early reproductive phase (DR-TS), which shortened the period of time available under favourable conditions prior to flowering (TS-AN), the dwarf lines produced fewer (23%) fertile florets per spike than that of the tall lines in the SS experiment. The decreased duration of late reproduction phase pre-anthesis (thermal time accumulated) was highly correlated with the reduced number of fertile florets (R^2^ = 0.908) in the SS experiment. There were more fertile florets in the top three spikelets of dwarf plants in both experiments (Table 5), suggesting that *Rht12* had a stronger production potential in the top spikelets. There was no significant difference between BB and bb in floret fertility.

Dry matter partitioning to the spikes during the late reproduction phase is particularly important for yield because during this phase the number of fertile florets at anthesis is determined [26]. In the AS experiment, the dry weights of the main shoot spikes at anthesis were similar among the four genotypes (Table 6). Whereas, the dry weight of the tiller spikes per plant in dwarf groups were higher (9%) than that of the tall groups, which might result from the increased number of tiller spikes in dwarf plants rather than bigger tiller spikes. Because of the reduction in plant stature, the dry weight of the main shoot (SDM) and the dry weight of the above-ground biomass (TDP) of the dwarf lines at anthesis were significantly decreased. While, the ratios of main shoot spike dry weight (SDM) to main shoot dry weight (PDM) and the total spike dry weight (SDM+SDT) to plant dry weight (TDP) of the dwarf lines was significantly increased compared to that of the tall lines (Table 6). This indicates that *Rht12* could increase the partitioning of dry matter to spikes during the late reproductive phase, potentially advantageous in achieving a greater availability of assimilates to support floret survival in developing spikes and a greater mass per carpel at anthesis, resulting in the greater production of fertile florets. But, in the SS experiment, SDM, SDT, and SDM/PDM of the dwarf genotypes at anthesis were all significantly lower than that of the tall genotypes, while (SDM+SDT)/TDP was not different between the dwarf and tall groups, and a fertility reduction was observed in the dwarf plants. It seemed that the environment effects of sowing date were greater on dwarf lines than on tall lines, and the differences between the two sowing date experiments were more evident in dwarf lines though all lines produced less total dry matter in the SS experiment than in the AS experiment.

**Table 6:** Dry weight of spikes and the whole plant at anthesis of different groups of the F_2:3_ lines in the autumn-sown (AS) and spring-sown (SS) experiments.

Experiment	Genotype/variety	SDM (g)	SDT (g)	PDM (g)	TDP (g)	SDM/PDM	(SDM+SDT)/TDP
AS	RRBB	0.71±0.05a	5.17±0.54a	3.16±0.32b	25.28±1.95b	0.22±0.01a	0.23±0.02a
	RRbb	0.70±0.04a	5.20±0.58a	3.05±0.38b	26.14±1.88b	0.23±0.02a	0.22±0.01a
	rrBB	0.71±0.06a	4.83±0.62b	3.95±0.46a	37.09±3.10a	0.18±0.01b	0.15±0.03b
	rrbb	0.71±0.04a	4.72±0.68b	3.77±0.41a	38.78±2.85a	0.19±0.01b	0.14±0.02b
	Karcagi	0.64±0.03	4.62±0.36	2.93±0.35	22.55±1.55	0.22±0.01	0.23±0.01
	Nchun45	0.88±0.05	4.17±0.30	4.18±0.35	33.68±2.30	0.21±0.02	0.15±0.01
SS	RRBB	0.64±0.04b	1.30±0.25b	3.45±0.30b	10.24±1.85b	0.19±0.01b	0.19±0.01a
	RRbb	0.62±0.03b	1.30±0.19b	3.38±0.35b	10.62±1.56b	0.18±0.01b	0.18±0.01a
	rrBB	0.85±0.06a	1.90±0.30a	4.10±0.54a	15.41±2.27a	0.21±0.02a	0.18±0.02a
	rrbb	0.83±0.06a	1.80±0.25a	3.95±0.50a	14.88±2.58a	0.21±0.02a	0.18±0.01a
	Karcagi	0.46±0.03	0.53±0.12	2.74±0.26	7.11±1.13	0.17±0.01	0.14±0.01
	Nchun45	0.91±0.03	2.00±0.18	4.79±0.45	17.31±1.58	0.19±0.01	0.17±0.01

Note: SDM is spike dry weight of the main shoot, SDT is spike dry weight of the tillers, PDM is the dry weight of the main shoot, TDP is the total dry weight of one plant. Data are means ± SD of each genotype. Data of the two parents were not considered in the statistical significance testing. Different letters within columns indicate statistically significant differences (*P*<0.05).

Furthermore, in the AS experiment, spikelet dry weight and number of fertile florets per spikelet of dwarf lines increased by, respectively, 2% and 13% compared with tall lines. Conversely, in the SS experiment, spikelet dry weight and number of fertile florets per spikelet of tall lines were larger than that of dwarf lines by 28% and 27%, respectively (Table 5 and Table 6). Despite these contrasting responses, in both experiments spikelet dry weight was highly correlated with the number of fertile florets per spikelet at anthesis with R^2^ of 0.81 and 0.99 respectively, in the AS and SS experiment.

### Plant Height and Associated Traits

**Figure 3:**
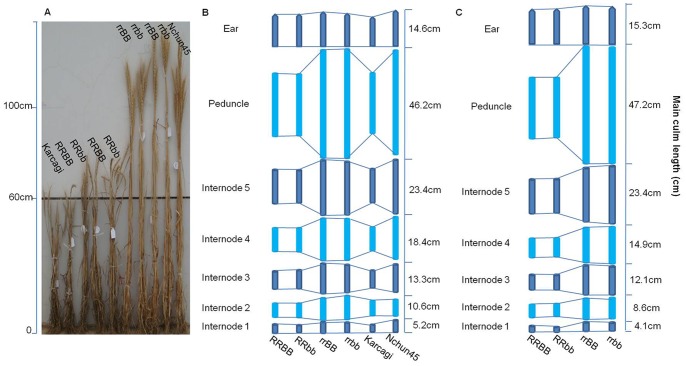
Culm morphology of the four genotypes in the AS and SS experiments. A: the mature plant morphology of the four genotypes and the two parents in the AS experiment. B: schematic representation of internode elongation patterns of the four genotypes and the two parents in AS experiment. C: Schematic representation of internode elongation patterns of the four genotypes in the SS experiment.

Plant height was greatly reduced by the *Rht12* dwarfing gene. In the AS experiment, the plant height of dwarf lines carrying *Rht12* was reduced by 45 cm (37%) through reducing the length of internodes, and the peduncle length was reduced by 20 cm (43%) in dwarf lines compared with that of the tall lines (Figs 3A, 3B and Table S1). In the SS experiment, plant height was reduced by 50 cm (40%) in the dwarf lines compared with that of the tall lines with the reduction of peduncle length by 23 cm (48%). The pattern of reduced internode length compared with the tall genotypes was thus similar in both the AS and SS experiments (Fig. 3C and Table S1).

**Figure 4:**
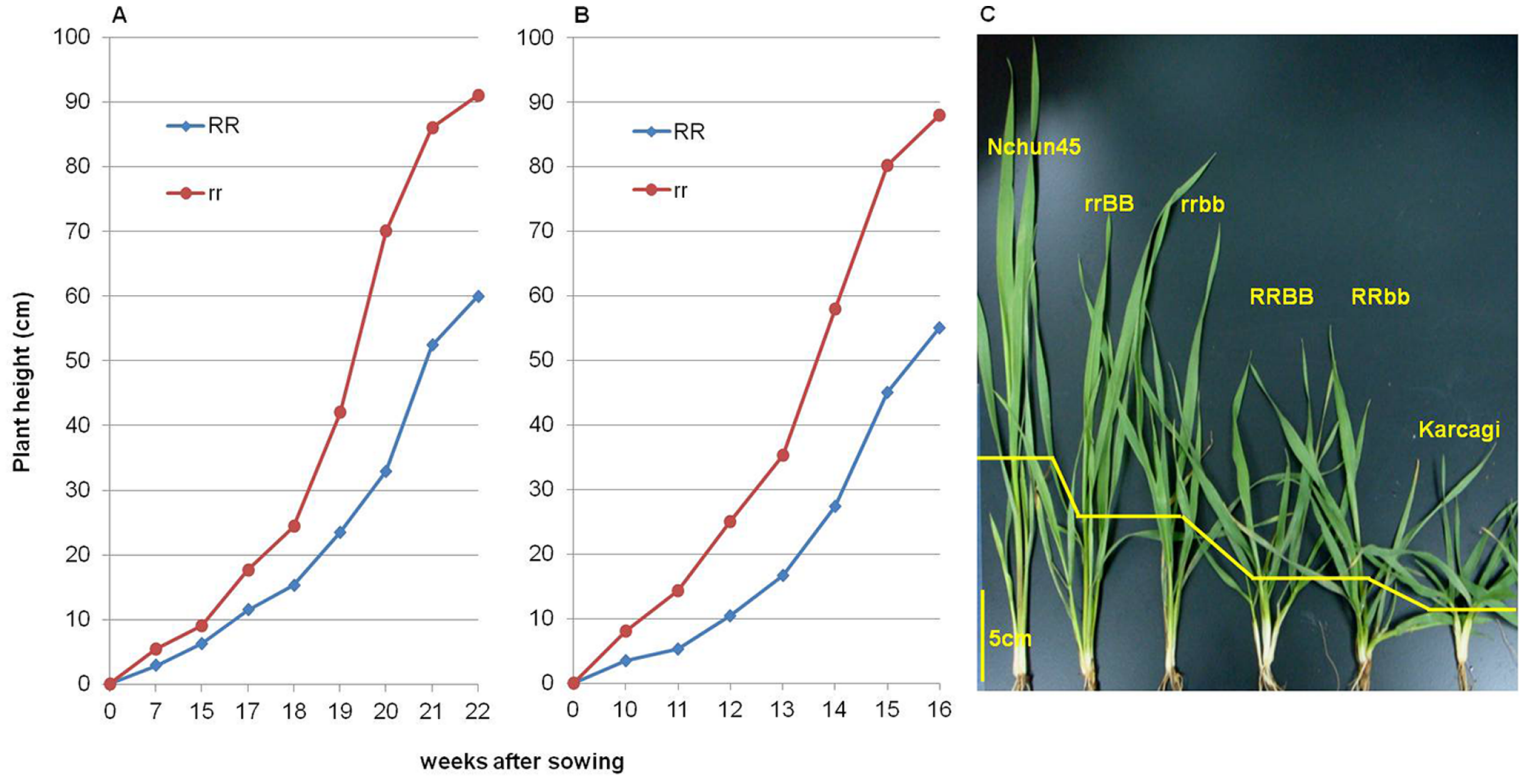
Development of plant height from the soil surface to the top ligule. A: AS experiment, the final height is achieved at week 22. B: SS experiment, the final height is achieved at week 16. C: plants at the 7^th^ week in AS experiment, the yellow line shows the site of the top ligule in each genotype and the two parents.

The culm elongated faster in tall lines than in dwarf lines from seedling stage (Fig. 4), with the tall lines reaching jointing stage earlier than the dwarf lines and producing longer internodes and more biomass; this difference was sustained to maturity in both experiments. The shortest individual internode was more often observed in RRbb lines in both AS and SS experiments. However, there was no significant difference in the length of internodes between BB and bb lines (Table S1). Although no significant difference was observed in diameter of the internodes between dwarf and tall lines, the thickness of the internode walls of the dwarf lines was significantly greater than that of tall lines in both experiments (Table S2). In particular, the wall thickness of the first and second internodes of the dwarf lines was increased by 0.25 mm (32%) and 0.22 mm (34%) in the AS experiment and 0.26 mm (28%) and 0.25 mm (31%) in the SS experiment, respectively. These shorter and thicker internodes might confer a greater resistance to lodging in the dwarf plants [57]. Due to the occurrence of storms at anthesis stage and at the middle grain filling stage, lodging occurred twice in the AS experiment; serious lodging was only observed in the tall lines (average lodging score is 39, Table S2), while all of the dwarf lines kept an upright posture. There was no significant difference observed in lodging between BB and bb lines.

There was no significant difference in the number of green leaves (average of 3.6) on main stem at anthesis between the four genotypes in both experiments. Whereas, the leaf length of dwarf lines with *Rht12* was significantly decreased (Table S3). In the AS experiment, the leaves of the dwarf lines were significantly shorter than those of the tall lines by as much as 6 cm (30%), and their widths were greater (10%∼25%) than those of the tall lines. Thus the total area of the uppermost three leaves on the main stem of the dwarf lines was only slightly (3%) less than that of the tall lines (Table S3). This may enable the dwarf lines to have a similar ability to produce sufficient photosynthate for the development of the kernels. Additionally, in both sowings, RRBB lines had significantly larger leaf area (4% and 6%, respectively) than RRbb whereas there was no effect of *Vrn-B1* in tall lines, suggesting that the dominant *Vrn-B1* could promote leaf growth in dwarf lines.

### Dry Matter Accumulation and Distribution of the Main Shoot after Anthesis

**Figure 5:**
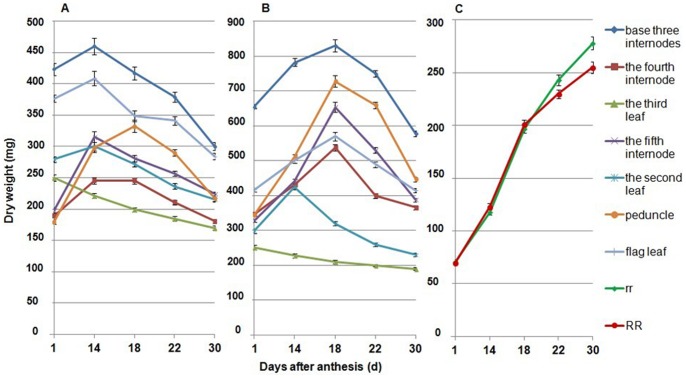
Dynamic changes in dry weight of different organs in the main shoot after anthesis in the AS experiment. A: dwarf lines; B: tall lines; C: spike dry weight of the dwarf and tall lines. The main culm comprises 6 internodes, the sixth internode is the peduncle. The leaves are numbered from the flag leaf down on the main stem. RR: dwarf lines; rr: tall lines.

**Figure 6:**
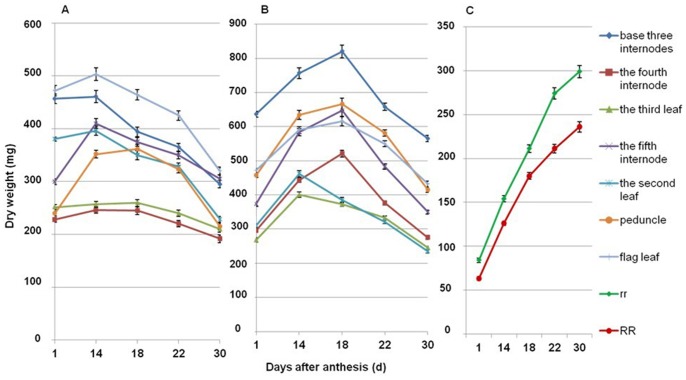
Dynamic changes in dry weight of different organs in the main shoot in the SS experiment. A: dwarf lines; B: tall lines; C: spike dry weight of the dwarf and tall lines. The main culm comprises 6 elongated internodes, the sixth internode is the peduncle. The leaves are numbered from the flag leaf down on the main stem. RR: dwarf alleles; rr: tall alleles.

Dynamic changes in the dry weight of different organs in the main shoot after anthesis were different between the dwarf and tall genotypes. In the AS experiment, the dry weight of the flag leaf, the second last leaf, the fourth internode and the fifth internode increased until the 14^th^ day (18^th^ day for peduncle) after anthesis in the dwarf lines, while the dry weight of the leaves and internodes were all increased until the 18^th^ day after anthesis, except the dry weight of the second last leaf, in the tall lines. The dry weight of the third last leaf decreased after anthesis in both dwarf and tall lines (Fig. 5 and Table S4). However, assimilates stored in leaves and stems of dwarf lines were transferred to spikes earlier than in the tall plants (Fig. 5). Similar results were also observed in the SS experiment on the dynamic change of dry weight of the leaves and stems (Fig. 6 and Table S5). In both AS and SS experiments, the maximum dry weights of the flag leaf were larger than that of the peduncle or the fifth internode in the dwarf plants while the opposite was observed in the tall plants. This indicated that *Rht12* affected internode growth more than leaf expansion. Finally, the total dry weight of the main shoot (excluding the spike) decreased by 0.67 g (30%) from its maximum to that measured at 30 days after anthesis in dwarf lines while it decreased by 1.37 g (35%) in tall lines in AS experiment; in the SS experiment the dry weight decreased by 0.87 g (33%) and 1.61 g (39%) in dwarf and tall lines, respectively. This suggests that the capability to support grain-filling (translocate shoot dry matter to the ear) was greatly reduced for dwarf lines, a conclusion supported by the observed greater gain in spike dry weight of tall lines (Figs. 5C and 6C).

### Yield and Yield Components

Seed set and grain numbers were significantly increased in *Rht12* dwarf lines compared to that in tall lines in the AS experiment, while fewer grains per spike were produced in dwarf lines under the conditions encountered in the SS experiment due to lower fertility (Table 7). For both sowings, there were more grains in the top three spikelets due to their higher floret fertility in dwarf lines than in tall lines (Tables 5, 7). This suggested that *Rht12* could increase grain numbers at the top of spikes and the top three spikelets in dwarf lines had stronger production potential than in tall plants. There was no significant difference between BB and bb lines for grain numbers.

**Table 7:** Effects of *Rht12* on yield components and harvest index of different groups of the F_2:3_ lines in the autumn-sown (AS) and spring-sown (SS) experiments.

Experiment	Genotype/variety	Grain number spike^−1^	1000-grain weight (g)	Number of efficient spikes plant^−1^	Plant yield* (g)	Plant biomass* (g)	Harvest index
AS	RRBB	45.8±3.95a	31.9±3.37b	15.4±1.24a	12.4±2.32a	35.6±6.86b	0.35±0.02a
	RRbb	44.6±3.46a	32.5±3.17b	14.8±1.73a	12.8±2.31a	35.3±6.13b	0.36±0.03a
	rrBB	41.6±3.63b	43.2±4.58a	12.6±2.19b	13.2±2.72a	42.6±8.29a	0.31±0.03b
	rrbb	40.8±3.64b	43.0±4.47a	13.4±1.42b	13.1±2.08a	42.5±6.79a	0.31±0.04b
	Karcagi	43.0±3.99	29.5±2.75	15.2±2.32	12.1±2.13	33.6±7.67	0.36±0.02
	Nchun45	45.4±4.34	50.3±3.02	10.4±1.28	13.5±2.01	40.8±8.50	0.33±0.03
SS	RRBB	36.3±2.59b	30.0±3.14b	2.6±0.50b	3.7±0.46b	11.8±2.56b	0.32±0.01a
	RRbb	34.1±2.88b	30.5±3.10b	2.2±0.36b	3.6±0.54b	12.5±2.63b	0.30±0.02a
	rrBB	47.4±4.21a	36.7±3.71a	4.0±0.87a	5.7±1.24a	20.9±5.15a	0.28±0.03b
	rrbb	46.7±3.29a	35.2±3.53a	3.5±0.62a	5.1±1.32a	19.1±3.83a	0.26±0.02b
	Karcagi	31.0±1.78	28.0±3.25	1.9±0.23	1.9±0.46	8.4±0.89	0.23±0.01
	Nchun45	50.1±4.26	37.2±3.16	4.3±0.27	6.3±1.59	21.5±2.13	0.29±0.02

* These data are the mean values of 25∼40 plants. All data are means ±SD of each genotype. Data of the two parents were not considered in the statistical significance testing. Different letters within columns indicate statistically significant differences (*P*<0.05).

A significant reduction in 1000-grain weight was found in *Rht12* dwarf lines compared to that in tall lines in both AS and SS experiments, possibly due to a combination of the 5-day delay in time to anthesis and the reduced plant biomass associated with *Rht12*. Whereas, the increase in grain numbers and in the number of fertile ears per plant of the dwarf lines resulted in there being no significant difference in plant yield between the dwarf and tall lines (Table 7). Additionally, as reported above, biomass per plant was decreased significantly in the dwarf genotypes, resulting in a net increase in harvest index. This indicates that, if total biomass could be maintained, *Rht12* might have the potential to decrease plant stature while increasing the grain yield. In the SS experiment, due to the lower fertility and less productive tillers of the dwarf lines, the grain yield of dwarf lines was much lower (32%) than that of the tall plants (Table 7). However, again due to the much-reduced biomass, the harvest index of the dwarf plants was higher than in the tall plants. This suggested that the dwarf genotypes could achieve a higher harvest index more easily than the tall genotypes in different environments. There was no significant difference between BB and bb lines on plant biomass, 1000-grain weight or grain yield.

## Discussion

This study is part of a series of experiments carried out to investigate and obtain a better understanding of the effects of the dwarfing gene *Rht12* on physiological attributes of the wheat crop. To improve the winter habit and the late ear emergence of *Rht12* plants [2], the dominant vernalization gene *Vrn-B1* was introduced to compensate for these effects. Contrasting homozygous lines with or without *Rht12* and *Vrn-B1* genes (RRBB, RRbb, rrBB and rrbb) were selected in a F_2_ segregating population and assessed as F_2:3_ lines to evaluate the effects of the dwarfing gene *Rht12*. Although the homozygous F_2:3_ lines do not share a common background as with near-isogenic lines (NILs), single chromosome recombinant inbred lines (RILs) or doubled haploid (DH) lines, the large number of lines studied allowed the effects of other loci to be reduced or neutralized, which coupled with the strong phenotypic effect of *Rht12*, gave good resolution between genotypes. A similar strategy could be used to assess the effects of other dwarfing genes with strong effects on plant height (such as *Rht4*, *Rht5*, *Rht13* and *Rht18*) to evaluate their potential use in crop breeding for improving agronomic performance or to determine their effects in different target environments.

To better understand the effects of *Rht12*, the F_2:3_ lines were sown at two sowing dates to provide winter and spring wheat growing environments, which was important for determining the spike development traits associated with *Rht12*. In the AS experiment, all plants should have received adequate vernalization, as around 6 weeks below 5°Cd are considered sufficient to complete vernalization of most wheat cultivars [58]. However, RRbb and even the RRBB group showed a longer duration to double-ridge and flowered later than the rrbb group. Moreover, in the SS experiment, rrBB and even rrbb reached the double ridge stage earlier and showed a higher ear fertility (than in the AS experiment) than RRBB, which still had a significantly elongated duration to double ridge and showed poor ear fertility. Therefore, the effects of *Rht12* on reduction in the rate of spike development and elongation of the duration of spike development phase, as well as its epistatic effects on *Vrn-B1* were revealed in the two sowing date experiments. This finding is different than that of the GA-insensitive *Rht* dwarf genes which had no effect on the duration of phenological development and reduced only the rates of vegetative and reproductive development [37], [38]. The effects of *Rht12* measured in the two experiments are thus very similar except for ear fertility and effective tiller number. The difference in ear fertility and effective tiller number between sowings could be the result of the elongated duration of plant development associated with *Rht12*: due to the slower growth rate and the strong winter growth habit caused by the strong effect of *Rht12*, also interacting with the higher temperature after the initiation of terminal spikelet, the dwarf plants had a shorter period of time under favourable conditions to develop fertile florets prior to flower [2], [26], and finally produced fewer competent florets than tall plants in the SS experiment. Additionally, the delayed elongation stage and the shorter time available to initiate new tillers and develop tiller spikes also decreased the number of tillers with fertile spikes after floret initiation stage in the dwarf lines. It is suggested that while floret development and effective tiller development were more sensitive to temperature than other traits in dwarf lines, it is feasible to evaluate other pleiotropic effects of *Rht12* using these two sowing date experiments under winter and spring growth conditions.

The reduction of plant height by *Rht12* was 47 cm (∼40%) in this study. A similar result was reported by Rebetzke et al. [21] who found that *Rht12* reduced plant height by around 44 cm (∼45%), which was stronger than the dwarfing genes most widely used in commercial varieties, such as *Rht1* (∼20%), *Rht2* (∼20%) or *Rht8* (∼7%) [21]. The reduction of plant height caused by these dwarf or semi-dwarf genes might result from reduced cell elongation rather than cell division [4], [34], [55]. Additionally, it was observed that while the diameter of the internodes was not affected by *Rht12*, the width of the stem wall was increased significantly in dwarf plants, which probably benefits lodging resistance [38]. Indeed, along with reduced height, thicker stem walls may contribute to the better lodging resistance of the dwarf plants in this study, and confirmed that dwarf genes had been an important factor in lodging resistance of commercial varieties [15], [17].

During the vegetative phase, the crop initiates leaves until the point of floral initiation, which is generally indicated by the formation of the first double ridge in the apex [26]. *Rht12* lines developed more leaves than the equivalent tall genotypes due to the longer duration to double ridge. In contrast, previous reports have shown that GA-insensitive dwarfing genes (*Rht1*, *Rht2* and *Rht3*) do not affect the timing of plant developmental events, nor the final numbers of leaves and internodes [3], [37], [38], [43]. This suggests that the two classes of dwarfing genes may act on plant growth and development via different mechanisms. While, similar with that in the GA-insensitive dwarf genotypes, the number of elongated internodes in *Rht12* lines did not change compared with the tall lines, indicating that *Rht12* affects the number of leaves but not the number of internodes that elongate. Despite initiating more leaves, there was no significant difference either in the number of green leaves at anthesis or the area of the three upper leaves at grain-filling stage between the dwarf and tall groups, which is important for radiation interception and biomass production in *Rht12* lines.

It is argued that the pre-anthesis late reproductive phase (TS-AN) coinciding with the period of rapid stem elongation, is particularly important for yield [25], [26] because the number of fertile florets at anthesis is determined during this phase [21], [59]. Moreover, it has been found that the greater number of fertile florets per spike at anthesis in dwarf lines than in tall lines is due to reduced degeneration of floret primordia in the late reproductive phase pre-anthesis rather than differences in the maximum number of floret primordia initiated [60], [61]. Lengthening the duration of TS-AN in wheat would arguably improve yield potential by increasing the number of fertile florets at anthesis [62], [63]. In the AS experiment, *Rht12* lines had a longer TS-AN duration (°Cd), which correlated with a significant increase in floret survival, although the total number of florets initiated was similar to the tall lines. The duration of TS-AN was also highly correlated with the number of fertile florets in the SS experiment, although in this case the dwarf lines actually had fewer fertile florets than the tall lines. Allocating a greater proportion of dry mass to the ear may also result in more fertile florets at anthesis [26], [64], but this does not imply that ears of dwarf genotypes are always heavier than those of tall genotypes, because total shoot dry matter has also to be considered [37]. With reduced competition due to a much shorter stem, *Rht12* could increase the partitioning of dry matter to spikes during the late reproductive phase, leading to a greater mass per carpel at anthesis, which in turn could result in an improved proportion of fertilized florets and finally produce more grains per spikelet and per ear.

Although the *Rht12* dwarf lines had a slower growth rate during stem elongation, the seeding vigour was similar to tall lines. The coleoptile length of dwarf plants was only about 3 mm (5%) shorter than that of the tall plants. Moreover, the length and width of the first leaves were not significantly different between the four genotypes. It has previously been observed that *Rht12* had no effect on seeding vigour and coleoptile length [21], [65], which offers the opportunity to reduce plant stature without compromising on early growth and crop establishment [20], [22]. In contrast, *Rht-B1b* or *Rht-D1b* are associated with shorter coleoptiles and smaller seedling leaf area and have a poor emergence when sown deeply [13].

It has been reported that *Rht12* had no negative effects on root dry mass [66]. In this study, a reduction in total number of seminal roots and a significant reduction in root dry mass (11%) were observed in *Rht12* dwarf lines at the seedling stage. These different findings may be due to the effect of either different genetic backgrounds or different growing environments, or both. In contrast, a longer maximum root length and a higher dry mass ratio of root to shoot were achieved in *Rht12* dwarf lines. Thus, *Rht12* had both negative and positive effects on root growth attributes. Further work should be conducted to explore the possibility of enhancing aspects of root growth such as root length using *Rht12* to enable crops to grow in drier environments.

The increase in ear fertility is observed as one the most important advantages of almost all the major height-reducing genes [3], [21], [37], [55]. Kernels per spike, especially the fertility of the top three spikelets were significantly increased in *Rht12* dwarf lines in the AS experiment. Consistent with that, *Rht12* significantly increased ear fertility in different varietal backgrounds, which results in the increase of grain numbers per spike [2]. Whereas, spike length, spikelet number per spike and (particularly) grain size were all reduced in the *Rht12* dwarf lines in this study as the lowest spike length and spikelet number per spike were observed in RRbb genotype while the highest in rrBB lines in both AS and SS experiments. Introduction of the dominant vernalization gene can help to compensate some of the effects of *Rht12* on yield parameters. Similar reductions in spikelet numbers have previously been correlated with genes other than height-reducing, particularly with *Ppd1*, where an average of two fewer spikelets per spike were produced [67].

The reduction in leaf length in lines containing dwarfing or semi-dwarfing *Rht-1* alleles (i.e. *Rht-B1b = Rht1*, *Rht-D1b = Rht2* and *Rhtb1c = Rht3*) is mainly due to shortening of cell length while the number of cells is not modified, and it has been suggested that *Rht-1* alleles may likewise limit cell length in the pericarp to reduce potential grain size [4]. However, Miralles et al. [55] found that *Rht-1* alleles did not modify cell length nor width in the pericarp, but reduced the total number of cells in that tissue, presumably by affecting cell division. It was also observed that the smaller average grain size conferred by *Rht* alleles were not only a consequence of a higher grain number produced in distal florets, but were also due to smaller grains at certain positions within the spike [3], [59]. Additionally, it has been suggested that reduced grain size with *Rht* alleles was associated with a competitive response to the increased spikelet fertility under limiting photosynthate availability, rather than a primary effect of the dwarfing genes [59]. Furthermore, flowering time can also influence grain size, because delayed flowering shortens the period of time available with favourable conditions prior to harvest [2]. In this study, the delay of 5–6 days in flowering time associated with *Rht12* could have affected the development and size of grains. Also, the small size of vegetative organs (especially the flag leaf and peduncle) of *Rht12* lines may have limited the assimilates available for producing large grains. Thus, the reduction in grain size of different dwarf genes may be through different modes of action from each other [6], [21], [55].

The pleiotropic effects of *Rht12* on several yield components were evaluated in the present study. *Rht12* increased the number of grains per spike and reduced grain size, resulting in an overall reduction in spike yield. Whereas, whole plant yields are dependent on both ear yields and the number of fertile ears produced per plant. Due to the increased number of tillers in dwarf plants (in AS experiment), the final yield per plant was not significantly different between the tall and dwarf plants. Although the plant biomass of dwarf plants was reduced, the harvest index of *Rht12* dwarf plants increased significantly in both AS and SS experiments. The greater harvest index and grain number for *Rht12* alleles was consistent with the effects of other dwarfing genes that reduced competition between growing florets and elongating stems [21]. *Rht12* has potential for increasing harvest index and total biomass in autumn sowing environment without compromising on establishment and early growth of seedlings, but it should be noted that in this study *Rht12* dwarf lines had lower yields in the spring sowing environment.

*Rht12* has been classified as a GA-responsive dwarfing gene [2], but its role, if any, in GA biosynthesis or signalling remains unknown. *Rht12* is located on chromosome 5AL, linked to Xgwm291 at a distance of 5.4 cM. Thus, high resolution mapping should be initiated for eventual map-based cloning of *Rht12*. To date none of the dwarfing genes are cloned except a few GA-insensitive dwarf genes [33]. More information on these genes is needed for a better understanding of how the dwarf genes act on plant growth and what their roles are in GA biosynthesis or signalling. However, it was found that the effects of the dwarf gene *Rht8* which had been considered as ‘GA-sensitive’ was possibly not due to the defective gibberellin biosynthesis or signalling, but possibly to a reduced sensitivity to brassinosteroids [34]. Similarly, there may be more complex relations between *Rht* genes and GAs uncovered by future research.

Previous research has indicated that the main disadvantage of *Rht12* is the long vegetative phase resulting in late ear emergence [2]. In this study, even the dominant *Vrn-B1*, which accelerates flowering, could not compensate for the negative effect of *Rht12* on flowering time. RRBB and RRbb had a similar duration of SW-DR while rrBB had shorter SW-DR duration than rrbb, suggesting that *Rht12* might be partially epistatic to *Vrn-B1*. Therefore, development-promoting genes need to be combined with *Rht12* to compensate for the delay in ear emergence in order to exploit the potential of *Rht12* in breeding programs. In this study, one dwarf line with earlier ear emergence time (2 days earlier than other dwarf lines) was observed, indicating that selection for early ear emergence may be possible among *Rht12* progeny. We have established other crosses for this purpose and also for further analysing the potential of *Rht12* for crop improvement.

## Supporting Information

Table S1 **Plant height and internode length of different groups of the F_2:3_ lines in the autumn-sown (AS) and spring-sown (SS) experiments.** All data are means ±SD of each genotype. Data of the two parents were not considered in the statistical significance testing. Different letters within columns indicate statistically significant differences (*P*<0.05).
(DOC)

Table S2 **Diameter and wall thickness of the internodes of different groups of the F_2:3_ lines in the autumn-sown (AS) and spring-sown (SS) experiments.** Note: DI is internode diameter at the mid-point; WT is wall thickness. The sixth internode is the peduncle. All data are means ±SD of each genotype. Data of the two parents were not considered in the statistical significance testing. Different letters within columns indicate statistically significant differences (*P*<0.05).
(DOC)

Table S3 **The mean length, width and area of the top three leaves of different groups of the F_2:3_ lines in the autumn-sown (AS) and spring-sown (SS) experiments at grain-filling stage.** *, The second and third leaves are from the flag leaf down on the main shoot. All data are means ±SD of each genotype. Data of the two parents were not considered in the statistical significance testing. Different letters within columns indicate statistically significant differences (*P*<0.05).
(DOC)

Table S4 **Dry weight (mg) of different organs in main shoot after anthesis of different groups of the F_2:3_ lines in the autumn-sown (AS) experiment.** *, The main culm comprises 6 internodes, the sixth internode is the peduncle. The leaves are numbered from the flag leaf down on the main stem. RR: dwarf alleles; rr: tall alleles. DDW is the difference between maximum dry weight and minimum dry weight, with its proportion to the maximum dry weight in the parenthesis. Values are given as the mean ±SD.
(DOC)

Table S5 **Dry weight (mg) of different organs in main shoot after anthesis of different groups of the F_2:3_ lines in spring-sown (SS) experiment.** *, The main culm comprises 6 internodes, the sixth internode is the peduncle. The leaves are numbered from the flag leaf down on the main stem. RR: dwarf alleles; rr: tall alleles. DDW is the difference between maximum dry weight and minimum dry weight, with its proportion to the maximum dry weight in the parenthesis. Values are given as the mean ±SD.
(DOC)
