# Supplementary material for: GA-Responsive Dwarfing Gene Rht12 Affects the Developmental and Agronomic Traits in Common Bread Wheat
Source: PLoS One. 2013 Apr 26;8(4):e62285. doi: 10.1371/journal.pone.0062285 (PMC3637298; doi:10.1371/journal.pone.0062285)
Supplement: Table S1 — Plant height and internode length of different groups of the F2:3 lines in the autumn-sown (AS) and spring-sown (SS) experiments. All data are means ±SD of each genotype. Data of the two parents were not considered in the statistical significance testing. Different letters within columns indicate statistically significant differences (P<0.05). (DOC) [file pone.0062285.s001.doc]

Table S1. Plant height and internode length of different groups of the F_2:3_ lines in the autumn-sown (AS) and spring-sown (SS) experiments

| Experiment | Genotype/ variety | 1^st^ internode | 2^nd^ internode | 3^rd^ internode | 4^th^ internode | 5^th^ internode | Penducle | Plant height |
| --- | --- | --- | --- | --- | --- | --- | --- | --- |
| AS | RRBB | 4.2±0.77ab | 6.2±0.66b | 7.9±1.20b | 10.0±1.05b | 14.5±1.98 b | 26.1±2.90b | 81.6±7.32a |
|  | RRbb | 3.4±0.65b | 5.9±0.40b | 7.8±1.41b | 10.4±1.01b | 14.3±2.02b | 26.1±2.96b | 80.1±7.61a |
|  | rrBB | 5.1±0.87a | 9.5±0.55a | 13.3±2.39a | 18.4±1.57a | 23.4±1.69a | 45.6±3.67a | 128.2±14.55b |
|  | rrbb | 4.5±0.87ab | 10.6±0.62a | 12.2±1.45a | 17.6±2.42a | 23.2±2.88a | 46.2±3.26a | 126.6±15.28b |
|  | Karcagi | 3.3±0.31 | 5.5±0.46 | 8.1±1.16 | 10.6±0.62 | 13.8±1.36 | 26.7±2.01 | 77.5±4.69 |
|  | Nchun45 | 5.2±0.62 | 6.9±0.41 | 12.4±1.25 | 17.7±0.83 | 23.2±1.12 | 43.9±2.36 | 122.8±5.58 |
| SS | RRBB | 2.7±0.77b | 5.4±1.05b | 6.4±0.77b | 7.9±0.97b | 14.1±1.36b | 24.7±3.29b | 74.6±4.11b |
|  | RRbb | 2.2±0.90b | 5.2±0.90b | 6.0±0.69b | 7.8±0.79b | 13.9±1.15b | 24.1±2.63b | 73.2±4.43b |
|  | rrBB | 4.1±1.15a | 8.6±1.20a | 12.1±1.32a | 14.2±1.07a | 22.9±1.81a | 47.2±4.21a | 124.4±13.69a |
|  | rrbb | 3.6±1.07a | 8.6±0.83a | 11.5±1.32a | 14.9±1.07a | 23.4±1.42a | 46.6±3.98a | 123.5±13.89a |
|  | Karcagi | 2.7±0.46 | 4.3±0.62 | 5.5±0.53 | 7.2±0.59 | 10.6±0.81 | 19.9±1.24 | 60.7±3.29 |
|  | Nchun45 | 4.8±0.66 | 9.1±1.04 | 12.2±1.08 | 15.4±1.05 | 21.1±1.36 | 44.7±1.24 | 119.0±5.93 |

All data are means ±SD of each genotype. Data of the two parents were not considered in the statistical significance testing.

Different letters within columns indicate statistically significant differences (*P* < 0.05).
