# Supplementary material for: GA-Responsive Dwarfing Gene Rht12 Affects the Developmental and Agronomic Traits in Common Bread Wheat
Source: PLoS One. 2013 Apr 26;8(4):e62285. doi: 10.1371/journal.pone.0062285 (PMC3637298; doi:10.1371/journal.pone.0062285)
Supplement: Table S2 — Diameter and wall thickness of the internodes of different groups of the F2:3 lines in the autumn-sown (AS) and spring-sown (SS) experiments. Note: DI is internode diameter at the mid-point; WT is wall thickness. The sixth internode is the peduncle. All data are means ±SD of each genotype. Data of the two parents were not considered in the statistical significance testing. Different letters within columns indicate statistically significant differences (P<0.05). (DOC) [file pone.0062285.s002.doc]

Table S2. Diameter and wall thickness of the internodes of different groups of the F_2:3_ lines in the autumn-sown (AS) and spring-sown (SS) experiments

| Expt | Genotype/variety | 1^st^ internode | | 2^nd^ internode | | 3^rd^ internode | | 4^th^ internode | | 5^th^ internode | | 6^th^ internode | | Lodging score |
| --- | --- | --- | --- | --- | --- | --- | --- | --- | --- | --- | --- | --- | --- | --- |
|  |  | DI (mm) | WT (mm) | DI (mm) | WT (mm) | DI (mm) | WT (mm) | DI (mm) | WT (mm) | DI (mm) | WT (mm) | DI (mm) | WT (mm) |  |
| AS | RRBB | 4.21±0.27a | 0.98±0.09a | 4.12±0.18a | 0.86±0.10a | 4.18±0.23b | 0.76±0.10a | 4.15±0.17a | 0.56±0.05a | 4.17±0.26a | 0.53±0.05a | 3.42±0.21b | 0.43±0.06a | 0.00±0.00b |
|  | RRbb | 3.96±0.24a | 1.06±0.10a | 4.21±0.17a | 0.87±0.11a | 4.15±0.22b | 0.74±0.09a | 4.07±0.15a | 0.55±0.04a | 4.14±0.25a | 0.51±0.06a | 3.42±0.25b | 0.42±0.05a | 0.00±0.00b |
|  | rrBB | 4.15±0.25a | 0.77±0.10b | 4.20±0.17a | 0.66±0.09b | 4.34±0.23a | 0.55±0.08b | 4.13±0.18a | 0.42±0.05b | 4.15±0.24a | 0.45±0.05b | 3.83±0.28a | 0.36±0.03b | 41.00±4.20a |
|  | rrbb | 4.11±0.22a | 0.78±0.10b | 4.23±0.19a | 0.63±0.11b | 4.30±0.21a | 0.56±0.09b | 4.05±0.20a | 0.41±0.06b | 4.13±0.24a | 0.44±0.05b | 3.80±0.29a | 0.34±0.05b | 37.00±3.85a |
|  | Karcagi | 3.85±0.18 | 1.10±0.08 | 4.06±0.18 | 0.87±0.07 | 4.09±0.18 | 0.71±0.06 | 4.02±0.12 | 0.58±0.04 | 4.15±0.18 | 0.56±0.05 | 3.27±0.15 | 0.42±0.03 | 0.00±0.00 |
|  | Nchun45 | 4.17±0.20 | 0.87±0.07 | 4.20±0.15 | 0.69±0.09 | 4.28±0.21 | 0.61±0.08 | 4.29±0.23 | 0.51±0.05 | 4.52±0.27 | 0.46±0.06 | 4.03±0.23 | 0.45±0.08 | 46.00±0.00 |
| SS | RRBB | 3.82±0.20a | 1.20±0.09a | 3.93±0.21a | 1.03±0.09a | 4.12±0.23a | 0.87±0.12a | 4.25±0.28b | 0.79±0.11a | 4.76±0.28b | 0.64±0.06a | 3.65±0.28b | 0.44±0.07a | 0.00±0.00b |
|  | RRbb | 3.75±0.21a | 1.19±0.11a | 3.87±0.20a | 1.03±0.09a | 3.94±0.24a | 0.89±0.11a | 4.23±0.27b | 0.80±0.11a | 4.71±0.27b | 0.62±0.05a | 3.66±0.29b | 0.44±0.06a | 0.00±0.00b |
|  | rrBB | 3.71±0.19a | 0.95±0.07b | 3.89±0.22a | 0.76±0.11b | 4.01±0.19a | 0.73±0.10b | 4.56±0.23a | 0.64±0.07b | 4.87±0.28a | 0.57±0.07b | 4.18±0.32a | 0.36±0.05b | 5.00±1.60a |
|  | rrbb | 3.79±0.17a | 0.92±0.08b | 3.90±0.20a | 0.81±0.12b | 4.23±0.25a | 0.71±0.10b | 4.54±0.25a | 0.63±0.07b | 4.92±0.30a | 0.57±0.06b | 4.15±0.30a | 0.37±0.05b | 10.00±1.80a |
|  | Karcagi | 3.40±0.11 | 1.18±0.07 | 3.64±0.16 | 0.94±0.10 | 3.73±0.21 | 0.83±0.08 | 3.90±0.19 | 0.74±0.13 | 4.21±0.33 | 0.58±0.09 | 3.17±0.25 | 0.33±0.06 | 0.00±0.00 |
|  | Nchun45 | 3.86±0.15 | 1.04±0.08 | 4.06±0.17 | 0.75±0.12 | 4.30±0.24 | 0.77±0.08 | 4.64±0.20 | 0.67±0.09 | 5.13±0.32 | 0.56±0.08 | 4.30±0.27 | 0.35±0.08 | 5.00±0.00 |

Note: DI is internode diameter at the mid-point; WT is wall thickness. The sixth internode is the peduncle. All data are means ±SD of each genotype. Data of the two parents were not considered in the statistical significance testing. Different letters within columns indicate statistically significant differences (*P* < 0.05).
