# Supplementary material for: GA-Responsive Dwarfing Gene Rht12 Affects the Developmental and Agronomic Traits in Common Bread Wheat
Source: PLoS One. 2013 Apr 26;8(4):e62285. doi: 10.1371/journal.pone.0062285 (PMC3637298; doi:10.1371/journal.pone.0062285)
Supplement: Table S3 — The mean length, width and area of the top three leaves of different groups of the F2:3 lines in the autumn-sown (AS) and spring-sown (SS) experiments at grain-filling stage. *, The second and third leaves are from the flag leaf down on the main shoot. All data are means ±SD of each genotype. Data of the two parents were not considered in the statistical significance testing. Different letters within columns indicate statistically significant differences (P<0.05). (DOC) [file pone.0062285.s003.doc]

Table S3. The mean length, width and area of the top three leaves of different groups of the F_2:3_ lines in the autumn-sown (AS) and spring-sown (SS) experiments at grain-filling stage

| Expt | Genotype/ variety | Flag leaf length (cm) | Flag leaf width | Flag leaf area (cm^2^) | Second leaf* length | Second leaf width | Second leaf area | Third leaf* length | Third leaf width | Third leaf area | Total leaf area |
| --- | --- | --- | --- | --- | --- | --- | --- | --- | --- | --- | --- |
| AS | RRBB | 15.6±1.35b | 1.9±0.09a | 23.7±2.04b | 20.9±1.96b | 1.7±0.08a | 28.4±3.21ab | 22.7±2.08b | 1.6±0.07a | 29.1±3.67a | 81.2±6.54a |
|  | RRbb | 14.8±1.40b | 1.9±0.08a | 22.5±2.10b | 20.1±1.92b | 1.7±0.08a | 27.4±3.30b | 21.9±2.12b | 1.6±0.08a | 28.0±3.87a | 77.8±6.78b |
|  | rrBB | 21.8±1.89a | 1.5±0.08b | 26.2±2.43a | 26.4±1.85a | 1.4±0.09b | 29.6±3.61a | 26.1±2.13a | 1.3±0.09b | 27.1±3.94b | 82.9±7.52a |
|  | rrbb | 21.4±1.82a | 1.5±0.08b | 25.7±2.45a | 26.8±1.90a | 1.4±0.08b | 30.0±3.66a | 25.6±2.06a | 1.3±0.08b | 26.6±3.62b | 82.3±7.44a |
|  | Karcagi | 15.2±1.18 | 1.8±0.05 | 21.9±1.56 | 21.1±1.53 | 1.5±0.07 | 25.3±1.67 | 22.0±1.74 | 1.4±0.07 | 24.6±1.96 | 71.8±3.89 |
|  | Nchun45 | 22.4±1.20 | 1.7±0.06 | 30.5±1.74 | 25.8±1.78 | 1.5±0.07 | 31.0±2.24 | 25.3±1.70 | 1.4±0.08 | 28.3±2.33 | 89.8±4.45 |
| SS | RRBB | 20.2±1.55b | 2.0±0.08a | 32.3±2.51b | 24.2±2.08b | 1.7±0.09a | 32.9±3.78a | 21.8±1.96b | 1.4±0.06a | 24.4±2.35a | 89.6±5.30a |
|  | RRbb | 20.5±1.62b | 1.9±0.08a | 31.3±2.60b | 24.8±2.10b | 1.5±0.06b | 29.7±3.60b | 22.9±1.92b | 1.3±0.06a | 23.8±2.20a | 84.7±5.16b |
|  | rrBB | 27.2±2.04a | 1.6±0.10b | 34.8±3.72a | 27.3±2.15a | 1.4±0.08b | 30.6±4.05b | 24.3±2.10a | 1.2±0.07a | 23.3±3.21a | 88.7±6.28a |
|  | rrbb | 26.8±2.01a | 1.7±0.09b | 36.4±3.53a | 26.6±2.22a | 1.4±0.08b | 29.7±3.95b | 24.8±2.05a | 1.2±0.08a | 23.8±3.17a | 90.0±6.11a |
|  | Karcagi | 19.0±1.34 | 2.0±0.04 | 30.4±1.74 | 24.4±1.63 | 1.5±0.05 | 29.3±1.85 | 21.1±1.33 | 1.0±0.05 | 16.9±1.25 | 76.6±3.35 |
|  | Nchun45 | 26.6±1.82 | 1.9±0.06 | 40.4±1.88 | 26.8±1.60 | 1.6±0.08 | 34.3±2.20 | 25.9±1.85 | 1.3±0.06 | 26.9±2.17 | 101.6±4.77 |

*, The second and third leaves are from the flag leaf down on the main shoot. All data are means ±SD of each genotype. Data of the two parents were not considered in the statistical significance testing. Different letters within columns indicate statistically significant differences (*P* < 0.05).
