# Supplementary material for: GA-Responsive Dwarfing Gene Rht12 Affects the Developmental and Agronomic Traits in Common Bread Wheat
Source: PLoS One. 2013 Apr 26;8(4):e62285. doi: 10.1371/journal.pone.0062285 (PMC3637298; doi:10.1371/journal.pone.0062285)
Supplement: Table S4 — Dry weight (mg) of different organs in main shoot after anthesis of different groups of the F2:3 lines in the autumn-sown (AS) experiment. *, The main culm comprises 6 internodes, the sixth internode is the peduncle. The leaves are numbered from the flag leaf down on the main stem. RR: dwarf alleles; rr: tall alleles. DDW is the difference between maximum dry weight and minimum dry weight, with its proportion to the maximum dry weight in the parenthesis. Values are given as the mean ±SD. (DOC) [file pone.0062285.s004.doc]

Table S4. Dry weight (mg) of different organs in main shoot after anthesis of different groups of the F_2:3_ lines in the autumn-sown (AS) experiment.

| Genotype | Days after anthesis | Base three internodes* | The fourth internode | The third leaf* | The fifth internode | The second leaf | Peduncle | Flag leaf | Main shoot spike |
| --- | --- | --- | --- | --- | --- | --- | --- | --- | --- |
| RR | 1 | 423.3±44.27 | 189.7±24.60 | 250.1±20.12 | 198.6±18.78 | 279.4±20.57 | 180.0±13.42 | 376.6±21.02 | 705.0±78.26 |
|  | 14 | 460.5±58.58 | 245.5±51.43 | 221.3±35.33 | 315.5±38.91 | 300.0±32.20 | 298.4±24.15 | 408.8±20.12 | 1230.1±131.03 |
|  | 18 | 417.2±45.17 | 245.1±36.67 | 199.1±42.48 | 281.0±24.15 | 272.3±17.89 | 332.7±20.57 | 348.6±16.10 | 2010.3±175.75 |
|  | 22 | 379.3±36.22 | 210.7±30.86 | 185.0±23.25 | 257.2±16.99 | 236.5±20.57 | 289.7±14.76 | 341.6±13.86 | 2300.0±180.22 |
|  | 30 | 300.2±29.07 | 180.6±23.70 | 170.1±20.12 | 224.4±15.65 | 215.4±15.20 | 217.5±14.31 | 284.1±14.31 | 2550.2±252.22 |
|  | DDW | 160.3(35%) | 64.9(26%) | 80(32%) | 116.9(37%) | 84.6(28%) | 152.7(46%) | 124.7(31%) | - |
| rr | 1 | 654.9±29.52 | 346.3±20.12 | 251.3±20.57 | 328.3±23.25 | 298.6±19.68 | 344.9±29.52 | 416.8±28.17 | 710.0±79.60 |
|  | 14 | 780.0±67.08 | 430.6±38.01 | 228.3±35.33 | 440.8±33.54 | 425.1±22.81 | 510.4±40.25 | 500.0±26.83 | 1180.0±126.11 |
|  | 18 | 830.0±76.92 | 538.0±73.79 | 210.0±65.29 | 653.9±55.90 | 320.0±44.27 | 727.8±30.41 | 570.0±25.49 | 1970.1±177.79 |
|  | 22 | 750.0±43.83 | 400.0±41.14 | 200.0±38.46 | 530.0±36.22 | 260.0±31.75 | 660.0±24.15 | 490.0±16.99 | 2430.2±225.84 |
|  | 30 | 577.6±36.22 | 365.9±33.09 | 190.0±25.04 | 387.7±29.07 | 230.0±26.83 | 446.8±21.47 | 415.0±18.34 | 2780.0±271.45 |
|  | DDW | 252.4(33%) | 191.7(36%) | 61.3(24%) | 325.6(50%) | 195.1(46%) | 382.9(53%) | 155.0(27%) | - |

*, The main culm comprises 6 internodes, the sixth internode is the peduncle. The leaves are numbered from the flag leaf down on the main stem. RR: dwarf alleles; rr: tall alleles. DDW is the difference between maximum dry weight and minimum dry weight, with its proportion to the maximum dry weight in the parenthesis. Values are given as the mean ±SD.
